# Quantitative evaluation of IVL: based on the tunica media gypsum calcification model and a prospective animal study

**DOI:** 10.3389/fcvm.2025.1620232

**Published:** 2025-07-11

**Authors:** Zongwei Liu, Chuang Xu, Bin Zhao, Jiayin Guo, Jingzhong Song, Jiaxue Bi, Yujun Shen, Xiangchen Dai

**Affiliations:** ^1^ Department of Vascular Surgery, Tianjin Medical University General Hospital, Tianjin, China; ^2^ Tianjin Key Laboratory of Precise Vascular Reconstruction and Organ Function Repair, Tianjin Medical University General Hospital, Tianjin, China; ^3^ Department of Pharmacology, Tianjin Key Laboratory of Inflammation Biology, Key Laboratory of Immune Microenvironment and Disease (Ministry of Education), School of Basic Medical Sciences, Tianjin Medical University, Tianjin, China; ^4^ Department of Research & Development Center, Sonosemi Medical Co., Ltd., Shenzhen, China

**Keywords:** intravascular lithotripsy, eccentric calcification, swine model, endovascular treatment, gypsum model

## Abstract

**Background:**

Intravascular lithotripsy (IVL), an emerging adjunctive therapeutic modality, demonstrates potential in managing severely calcified lesions. However, its quantitative efficiency in disrupting calcifications with different characteristics, as well as the degree of damage to normal arteries, remains to be confirmed.

**Objectives:**

This study aimed to: (i) quantitatively evaluate the efficacy of IVL in disrupting different types of calcifications, and (ii) assess the impact of IVL on normal vascular structures.

**Methods:**

The gypsum models with different thicknesses and eccentricities were used to evaluate the effectiveness of IVL in disrupting calcifications with different characteristics. *In vivo* experiments involved iliofemoral arterial segments of nine *Yorkshire* experimental swine that were subjected to IVL and PTA working balloons, respectively. *In vitro* effectiveness of IVL was evaluated using the number of disrupted gypsum rings based on the gypsum models. *In vivo* effectiveness and safety of IVL were evaluated by digital subtraction angiography (DSA), light microscopy, and immunofluorescence staining based on the experimental swine at 0, 7, and 28 days.

**Results:**

The gypsum models revealed that the 1.04:1 oversized IVL working balloon could provide an optimal tightness between the balloon and the artery wall. The DSA imaging results showed that IVL significantly increased the immediate treated artery's diameter at +27.12 ± 10.23% compared to the PTA working balloon at +13.72 ± 7.66% in all experimental animals (*n* = 9, *p* = 0.0063). The imaging results revealed that IVL treatment significantly alleviated the lumen loss rate of treated arteries compared to the PTA working balloon at 7 (1.10 ± 0.58% vs. 3.27 ± 0.66%) and 28 (4.90 ± 1.60% vs. 10.10 ± 1.53%) days postoperatively (*p* < 0.05). Histopathological analysis showed the IVL treatment did not increase the inflammatory status, synthesis of collagen, and other artery wall characteristics at 0, 7, and 28 days postoperatively. The immunofluorescence staining results revealed that IVL treatment did not significantly decrease the proportion of smooth muscle cells and endothelial cells in the treated artery.

**Conclusion:**

Our experiment revealed that the IVL device has good therapeutic effects on different characteristics of calcifications hiding in the tunica media and with good biological safety.

## Introduction

Lower extremity peripheral artery disease (PAD) affects over 200 million people worldwide, with symptoms ranging from asymptomatic presentations to critical limb ischemia (1). As the global population ages, the prevalence of PAD has surpassed 8.5 million cases in developed countries (2). Clinical manifestations may include varying degrees of lower limb pain, claudication, and ischemia-induced weakness. The 2024 American College of Cardiology/American Heart Association guidelines recommend tailored rehabilitation exercises and lipid-lowering drugs to alleviate lower limb discomfort and delay disease progression (3). However, in advanced PAD, endovascular revascularization often becomes the only option to prevent limb amputation. Although advancements in endovascular treatment (EVT) devices have mitigated challenges such as thrombosis and chronic total occlusion, severe calcification within the tunica media of peripheral arteries remains a major unresolved obstacle.

Calcified plaques are challenging to remove from lesions but can be redistributed along the arterial wall through balloon compression, improving arterial compliance and facilitating subsequent drug-coated balloon (DCB) or stent-graft implantation for revascularization. However, balloon overexpansion to compress hard calcified plaques increases the risk of arterial rupture. Current calcium modification techniques face two critical limitations: (i) Directional atherectomy removes calcified plaques by crushing and extracting them from the artery, but meta-analyses indicate that it may increase the risk of arterial rupture by 38% [relative risk: 1.38, 95% confidence interval (CI): 1.12–1.71] (4), and (ii) excimer laser ablation (ELA) is another method used to treat arterial calcification, which can generate ultraviolet light at a wavelength of 308 nm to disrupt soft lipid plaques and is commonly used to treat in-stent restenosis. However, insufficient clinical evidence in PAD patients limits the evaluation of its safety (5). Notably, both methods require direct contact with calcified lesions, making it difficult to address calcifications within the arterial tunica media. This limitation remains a critical challenge of EVT in patients with PAD.

To address this challenge, an effective and innovative endovascular device was developed to fracture calcified plaques without direct contact. Intravascular lithotripsy (IVL), adapted from ultrasound lithotripsy technology used in the urinary system, was first applied to treat calcified plaques in coronary arteries, achieving a procedural success rate of 98.0% in heavily calcified lesions (6, 7). The density difference between calcified plaques and normal blood vessels creates an impedance mismatch in their acoustic properties. As a result, tissues with higher density, such as calcified plaques, experience greater pressure from non-aggregated low-intensity shockwaves. The IVL device generates unfocused circumferential pulses through the electrohydraulic effect, enabling the disruption of calcified lesions without direct contact with the plaques (8).

Prospective clinical trials of IVL have demonstrated its potential to enhance the efficacy of EVT for calcified lesions (9–11). However, the efficiency of IVL in disrupting plaques with varying calcification characteristics and its potential impact on normal vascular walls remain a concern. To address these limitations, this study introduces a novel intravascular shockwave lithotripsy system (LiqMagic P18, Sonosemi Medical) specifically engineered to target tunica media calcifications. Through *in vitro* calcification models and *in vivo* validation in porcine models, the device demonstrated high efficacy and safety across heterogeneous calcification subtypes. Our experiments quantitatively demonstrated the disruption efficiency of the IVL device on different characteristic calcifications and the degree of damage to normal blood arteries. Thus, an IVL device may offer a reliable therapeutic option for treating severely calcified lesions of the tunica media.

## Methods

### Experimental design

**Figure 1:**
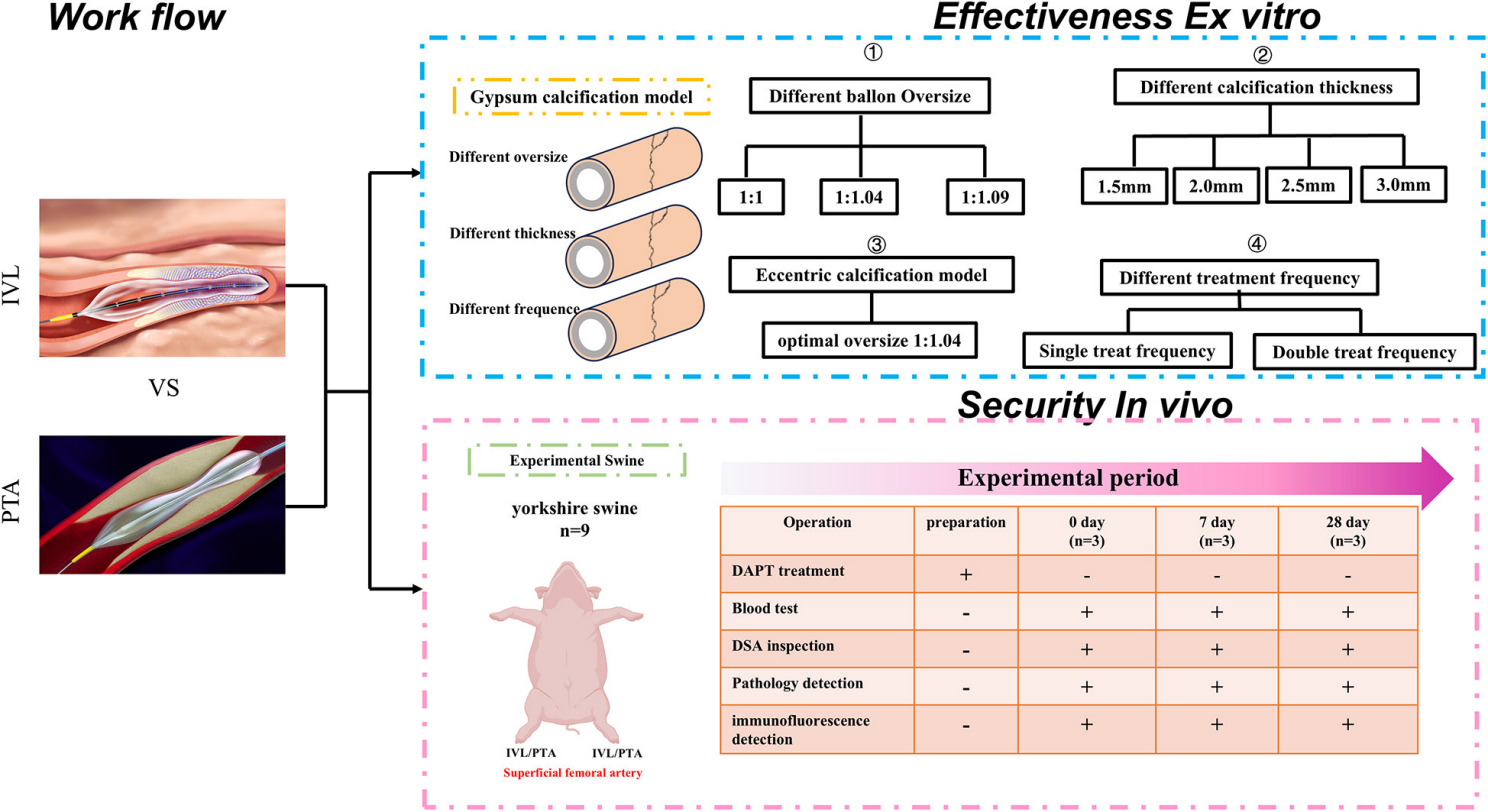
Flowchart.

A study flowchart is presented in Figure 1. The animal study was approved by the Institutional Animal Ethics Committee of the hospital.

### Animal experimental procedures

According to the previous study, female and male experimental animals were enrolled (12). For the animal study, nine Yorkshire swine (mean age: 12.3 ± 0.5 months; average weight: 41.0 ± 2.4 kg) were included. A total of one female and two male experimental animals were enrolled in each experimental group.

Before the procedure, the swine were housed individually in kennels measuring 2.5 m^2^ per animal, following ISO 10993-2 standards. The cages were ventilated and dry, with *ad libitum* access to food and water during a 14-day acclimatization period. The animals were fasted for 24 h and deprived of water for 12 h before surgery. The left and right iliofemoral arteries of each pig were treated with either IVL or percutaneous transluminal angioplasty (PTA; Sterling OTW, Boston Scientific Corporation, Natick, Massachusetts, USA).

Three swine were assigned to the acute group (0 days, *n* = 3) and euthanized immediately after the procedure. The remaining swine underwent follow-up assessments, including digital subtraction angiography (DSA) and physiological monitoring, and were euthanized at 7 days (*n* = 3) and 28 days (*n* = 3).

The experiment was conducted at an independent animal facility certified through a conformity assessment. Dual antiplatelet therapy (DAPT; 75 mg clopidogrel and 325 mg aspirin daily) and prophylactic antibiotics (0.5 g ceftriaxone sodium) were administered 3 days preoperatively and continued throughout the study according to the previous studies (13, 14). After sedation, all animals were intubated and maintained under general anesthesia with 1%–5% isoflurane inhalation. Vital signs were continuously monitored during the procedure using lead II electrocardiography and invasive arterial pressure measurement via carotid access.

Endovascular procedures were performed using a trans carotid approach with 6F introducer sheaths (Terumo Medical, Tokyo, Japan). Heparin (400 IU/kg) was administered intravenously before catheterization according to the previous study (15). Target arteries were selected based on the reference vessel diameter measured using quantitative vascular angiography to achieve a balloon-to-artery ratio of (1.0–1.1):1.0. The IVL balloon was inflated with a 30% iodixanol–saline mixture (Visipaque™, GE Healthcare) to a working pressure of 4 atm, monitored in real-time DSA inspection to ensure apposition to the arterial wall. After delivering 20 pulses at a frequency of 1 Hz, the balloon was deflated and held for 20 s to restore blood flow. This sequence was repeated for four cycles, delivering a total of 80 pulses—the maximum per IVL catheter. For PTA, the working balloon was placed in the contralateral iliofemoral artery with the same balloon-to-artery ratio of (1.0–1.1):1.0. The balloon was inflated for 20 s at nominal pressure, with inflation time extended to 30 s between cycles to align with clinical protocols. The PTA procedure was also repeated for four cycles.

### Intravascular shockwave lithotripsy system device

IVL differs from other peripheral artery debulking devices by generating circumferential low-intensity shockwave energy with pressures of 1.0 MPa for PTA at 10 atm and 2.8–9.7 MPa for IVL at 4 atm. This novel approach for treating calcified lesions allows interventional physicians to disrupt calcifications effectively and safely while minimizing damage to surrounding tissue, thereby improving arterial compliance for subsequent EVT. We used the IVL system included a LiqMagic P18 IVL working balloon (registration number NMPA20243012461, code number SI-SC002-5060) and an ISL200 shockwave generator（registration number NMPA20243012473, which was provided by Sonosemi Medical Co., Ltd. (Shenzhen, China).

**Figure 2:**
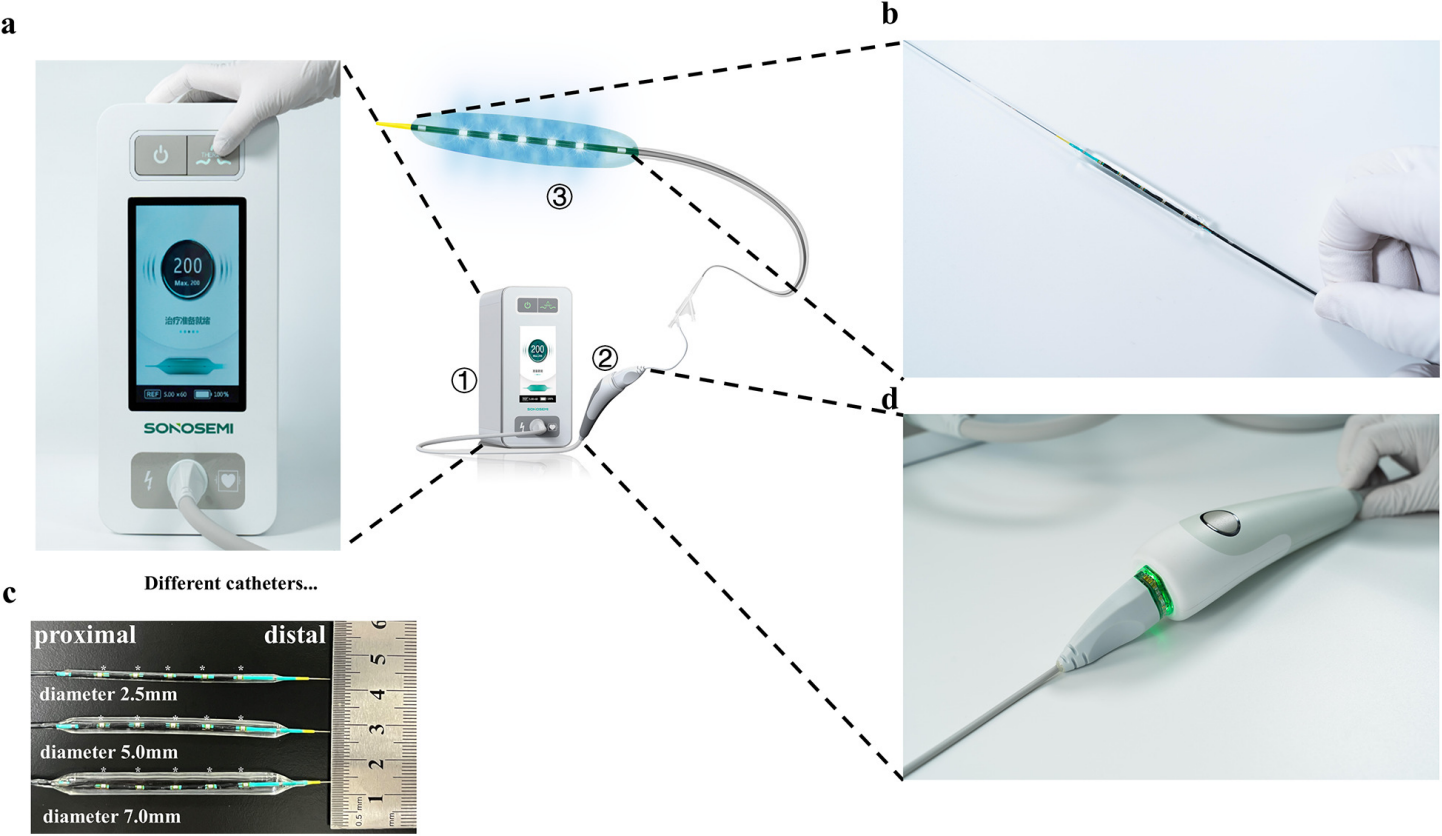
IVL device. **(a)** IVL battery and user interaction display screen; **(b)** electrohydraulic lithotripsy emitters and working balloons; **(c)** different specifications of working balloons, * marked the shockwave emitter positions; **(d)** IVL operating handle with only one operation button. IVL, intravascular lithotripsy. **(a)**, **(b)**, **(d)** Images provided by Sonosemi, https://www.sonosemi.com/.

The IVL system consists of three main components: (1) a generator, (2) IVL catheters, and (3) IVL connector cables. The circumferentially focused low-intensity shockwave generator is a fully portable, rechargeable unit (dimensions: 152 mm × 285 mm × 340 mm; weight: 5.8 kg) capable of delivering up to 3,000 V of electrical energy, generating small electrical sparks. Its treatment frequency is 1 pulse per s (1 Hz), with each treatment cycle consisting of 20 s of continuous operation, delivering 20 circumferential low-intensity shockwaves. This design ensures effective therapeutic outcomes while minimizing patient radiation exposure. The generator is powered by two long-lasting lithium batteries (12.8 Ah) and features a user-friendly liquid crystal display that provides clear information on the balloon catheter status, the remaining number of shockwaves, the current operating status, and the remaining battery capacity (Figure 2a).

The emitters convert electrical energy into transient acoustic circumferential pressure pulses that selectively disrupt both superficial and deep calcifications within the artery, enhancing vessel compliance while preserving the fibroelastic components of the vessel wall. The IVL system offers single-use balloon angioplasty catheters in various sizes (diameters ranging from 2.5 to 7.0 mm), each equipped with five unfocused electrohydraulic lithotripsy emitters. These catheters can withstand burst pressures of up to 12 atm (Figures 2b,c), allowing surgeons to select the optimal catheter based on lesion characteristics, with enhanced safety.

The catheter is compatible with a 0.018-inch guidewire system, commonly used for lower extremities, eliminating the need to change the guidewire for additional stenting or angioplasty procedures. A key feature of this IVL catheter is its five uniformly distributed, unfocused shockwave emitters, which release circumferentially focused low-intensity shockwave energy evenly across the balloon surface, ensuring uniform calcification disruption. The IVL connector cable is 1.6 m long and includes a start and interruption button, facilitating convenient and timely operation by interventional physicians (Figure 2d).

In summary, this IVL system is a novel, user-friendly endovascular device designed for convenient and adaptable treatment of various lesions.

### Identification of IVL effectiveness using an *in vitro* gypsum calcification model

**Figure 3:**
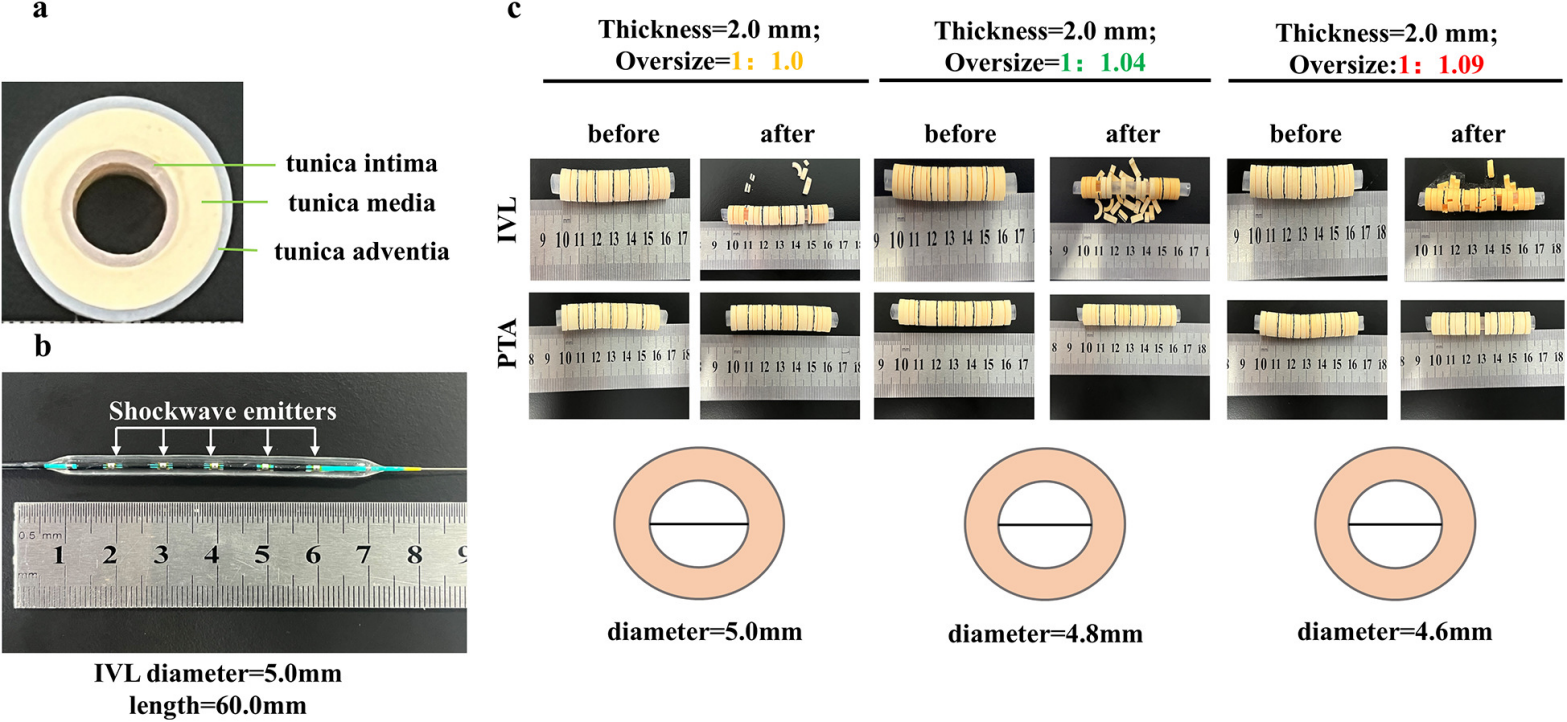
Validation of gypsum models at different oversize ratios. **(a)** customer-modified gypsum calcification model with 1-mm-thick silicone gel “tunica intima” layer, a 2-mm-thick gypsum layer representing the “tunica media,” and a 1-mm-thick silicone gel “tunica adventitia” layer; **(b)** treated IVL working balloon with five shockwave emitters; **(c)** different oversized treatments for calcified gypsum models to determine the optimal oversized ratio.

To validate the effectiveness of the IVL system for different degrees of calcification, customer-modified gypsum calcification models with varying thicknesses were constructed and modified from urological stone models (16). To simulate the characteristics of target lesions in patients with PAD, the gypsum calcification model included a 1-mm-thick silicone gel “tunica intima” layer, a 2-mm-thick gypsum layer representing the “tunica media”, and a 1-mm-thick silicone gel “tunica adventitia” layer (Figure 3a). A black marker on the gypsum rings indicated the position of the shockwave emission electrode.

According to a previous study (17), the tightness between the working balloon and vascular wall can affect the plaque disruption effectiveness of IVL. To determine the optimal working tightness, a 60 mm working catheter with a 5 mm diameter was tested, as it is commonly used in PAD procedures. To create models with varying tightness between the working balloon and the gypsum calcification, the inner layers of the gypsum model were constructed with different diameters (5.0, 4.8, and 4.6 mm). This design allowed for different oversized ratios of the working balloon to the gypsum ring (1:1, 1.04:1, and 1.09:1; Figures 3b–d).

### Angiographic analysis

All angiographic images were analyzed using RadiAnt software version 2022.1.1 (64-bit; Medixant, Poznan, Poland) by two independent interventional radiologists blinded to treatment allocation. The treated artery segments were matched across preoperative, postoperative, and terminal angiography images using bony landmarks (femoral head centroid), branch vessel origins (diameter ≥ 1 mm), and intravascular contrast patterns for alignment according to the previous study (15).

The following parameters were measured within the treated target segments: the reference artery diameter (RAD), defined as the maximum diameter of the target artery before treatment; the maximum diameter of the target lumen after treatment; and the minimum luminal diameter during the follow-up period. The lumen acquisition rate was calculated as follows: [(maximum diameter of the target lumen after treatment−RAD) ÷ RAD] × 100%. The late lumen loss rate was calculated as follows: (1−target artery minimum diameter during follow-up ÷ RAD) × 100%.

### Histological and immunofluorescence analysis

Histological changes were assessed using light microscopy and immunofluorescence staining. Specimens were harvested based on angiographic landmark matching and *in situ* observation. Microscopic specimens were immediately immersed in 10% neutral-buffered formalin for 24 h for fixation and then embedded in paraffin using standard procedures. The specimens were stained with hematoxylin and eosin, Masson's trichrome, and Verhoeff's van Gieson according to the instructions of the manufacturers.

For immunofluorescence staining, tissue sections were incubated overnight at 4°C with primary antibodies diluted 1:100 (smooth muscle protein 22-alpha, Proteintech #60213-1-Ig, validated for porcine tissue; CD31, Proteintech #11265-1-AP). After three washes with phosphate-buffered saline, the sections were incubated with secondary fluorescent antibodies [Goat anti-Rabbit IgG(H + L)-HRP, UTIBODY #UT2001; Goat anti-Mouse IgG(H + L)-HRP, UTIBODY #UT2003] for 1 h at 37°C in the dark. The sections were mounted with 4′,6-diamidino-2-phenylindole (ab104139; Abcam, USA) and examined using a fluorescence microscope (Leica, Germany).

### Microscopy analysis

Light microscopy was performed by a blinded pathologist. Specimens were cut into 4-µm sections and stained using standard procedures. Five random fields of view were selected for semi-quantitative statistical analysis, with scoring criteria as previously described (15). For the semi-quantitative analysis of immunofluorescence results, 90 images were obtained from five random fields within a 20× magnified field of view from both the experimental and control arteries of nine swine. The fluorescence signal area of the target channel was standardized using the fluorescence signal area of 4′,6-diamidino-2-phenylindole.

### Statistical analysis

To calculate the power calculation and justification for the sample size, we calculated the sample size using *a priori* power analysis software, GPower (University of Dusseldorf, Germany). To detect a 15% difference in mean acute lumen acquisition (α = 0.05, power = 0.80), a minimum of *n* = 9 animals/group was required with effect size d = 1.48. Our final sample size (*n* = 9) reached this threshold.

Continuous variables were expressed as mean ± standard deviation, and categorical variables were expressed as percentages. Continuous variables were analyzed using the *Student-t* test for comparisons between two matched groups. Statistical analyses were performed using GraphPad Prism 10 (GraphPad Software, Inc., La Jolla, California, USA). All probability values were two-tailed, and a *p*-value < 0.05 was considered statistically significant.

## Results

### Identification of IVL effectiveness using an *in vitro* gypsum calcification model

**Figure 4:**
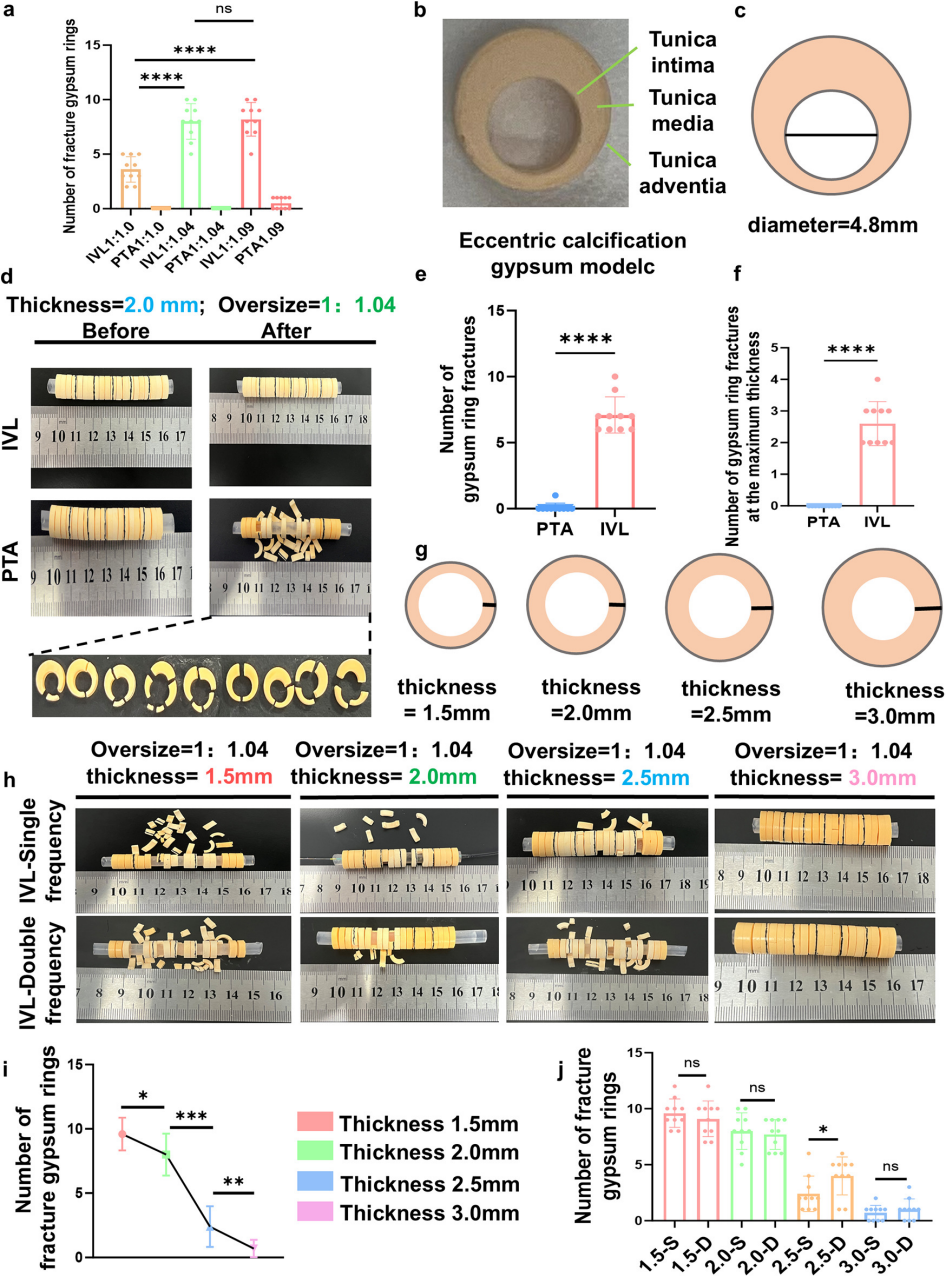
Effectiveness of the IVL in disrupting the calcification gypsum model. **(a)** different oversized ratios to identify the optimal working oversized ratio at the 1.04:1; **(b,c)** a customer-modified eccentric calcified gypsum model; **(d)** customer-modified eccentric calcified gypsum model disrupted by IVL working balloon; **(e,f)** number of fractures in eccentric calcified gypsum models using IVL working balloon with 1.04:1 optimal oversized ratio; **(g)** different calcification thicknesses to achieve a different oversized ratio treratment; **(h)** different calcification thicknesses gypsum model disrupted by single and double frequency treatment cycles; **(i,j)** results of single-frequency and double-frequency IVL treatment on gypsum models with different calcification thicknesses. IVL, intravascular lithotripsy.

A 2.0-mm “tunica media” gypsum calcification model was treated with four cycles at different oversize ratios. The number of disrupted gypsum rings was significantly higher with IVL compared with traditional PTA balloons (4.2 ± 1.1 vs. 1.3 ± 0.6 fractures/ring, *p* < 0.001). Specifically, the 1.04:1 oversized ratio, selected based on vascular biomechanical modeling, resulted in a significantly greater number of disrupted gypsum rings than the 1:1 ratio, whereas the 1.09:1 ratio did not show additional improvement over the 1.04:1 group (Figure 4a). These findings indicate that the IVL system is more effective than traditional PTA balloons for disrupting calcification at different oversize ratios, with the 1.04:1 ratio providing optimal tightness between the balloon and arterial wall for enhanced disruption.

Clinically, patients with PAD often present with diverse calcification characteristics, including eccentric calcification, which can result in uneven disruption by devices such as atherectomy or ELA. This may lead to suboptimal lesion preparation and an increased risk of adverse events.

To evaluate the efficacy of the IVL device in treating eccentric calcification, an eccentric calcification model was constructed (Figure 4b). Using a catheter with a 5.0 mm diameter and 60 mm length for four treatment cycles, the IVL system demonstrated superior performance compared with the PTA balloon in disrupting eccentric calcification at the optimal oversize ratio (1.04:1), as evidenced by a greater number of fractured gypsum rings at maximum thickness (3.8 ± 0.9 vs. 1.1 ± 0.4 fractures/section, *p* = 0.003; Figures 4c–f).

Another challenge is that calcification thickness significantly affects the effectiveness of target lesion preparation. To evaluate the therapeutic effects of the IVL system on calcified lesions of varying thicknesses, a gypsum calcification model was constructed with tunica media calcification thicknesses of 1.5, 2.0, 2.5, and 3.0 mm (Figure 4g). Under optimal oversize conditions, a catheter with a 5.0 mm diameter and 60 mm length was used to treat the different models over four treatment cycles. As expected, increasing calcification thickness reduced the effectiveness of the IVL device, as evidenced by a decreasing number of disrupted gypsum rings.

To counteract the weakening effect of increased calcification thickness on IVL-induced calcification disruption, an additional four treatment cycles were applied to previously treated calcified gypsum rings (Figures 4h–j). The results demonstrated that the additional treatment cycles restored IVL treatment efficacy, as evidenced by an increased number of disrupted gypsum rings.

Overall, the results from the gypsum ring calcification model suggest that this IVL device is effective in treating various types of calcifications, potentially improving target lesion preparation for subsequent EVT.

### An experimental *in vivo* swine study demonstrates the effectiveness and safety of the IVL device

In the *in vitro* experiment, the IVL system demonstrated greater effectiveness in disrupting calcification compared with the traditional PTA balloon. To validate the safety of the IVL system, an *in vivo* experiment was conducted using healthy 12-month-old Yorkshire swine with a mean weight of 41.0 ± 2.4 kg. Nine swine, regardless of sex, were enrolled and divided into three treatment groups corresponding to 0, 7, and 28 days. Basic information about these animals was summarized in (Supplementary Table S1).

**Figure 5:**
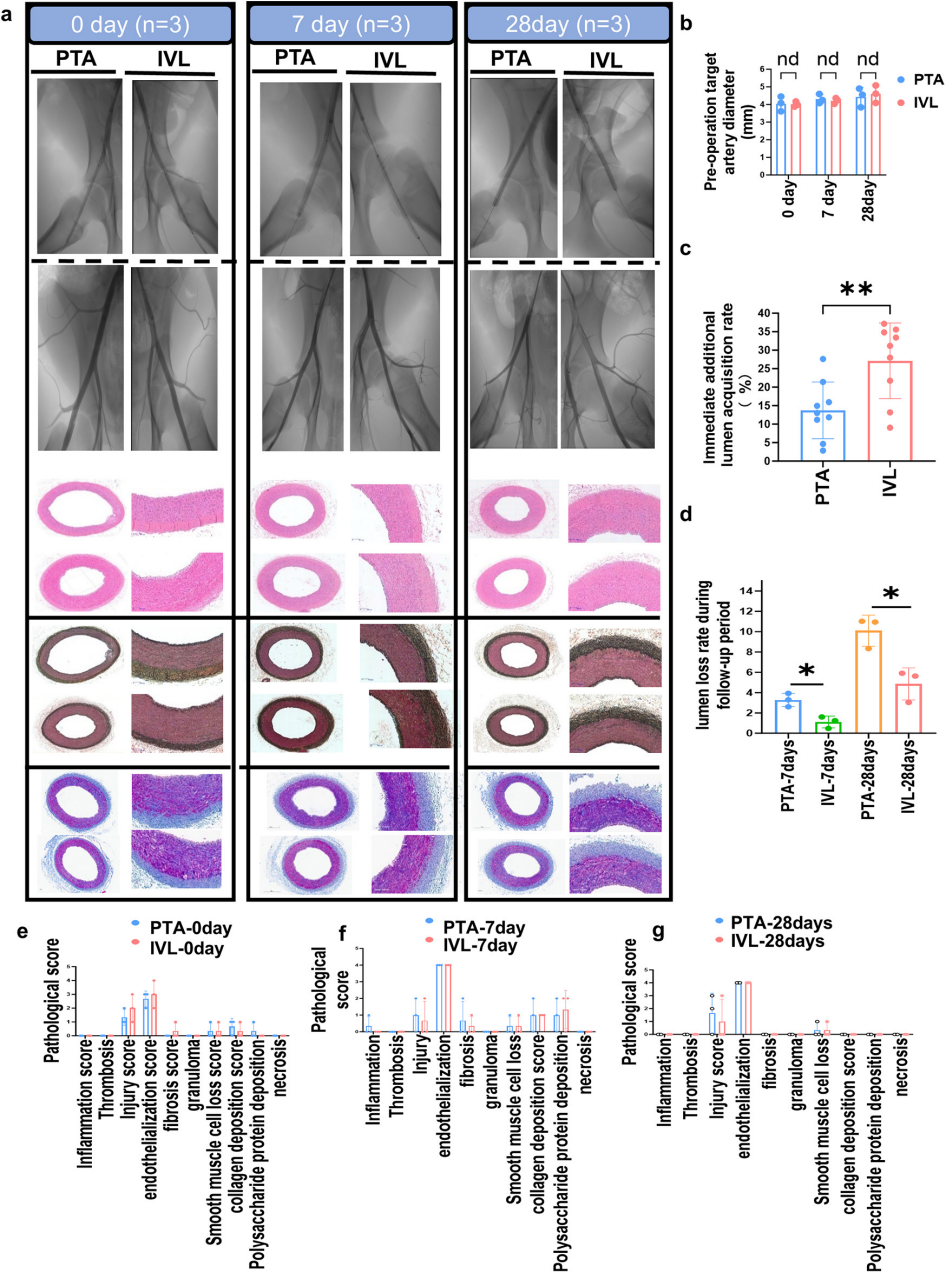
DSA imaging and histopathological analysis. **(a)** DSA imaging and histopathological sections during the experimental animal's follow-up period; **(b–d)** lunmen acquisition diameters calculation after IVL treatment; **(e–g)**: analysis of target artery pathological scoring. DSA, digital subtraction angiography.

To reduce selection bias, the bilateral superficial femoral arteries of each swine were randomly assigned using computer-generated randomization to either the experimental group (IVL treatment) or the control group (PTA treatment). Preoperative DSA imaging showed comparable target lesion characteristics between the two groups (Figure 5a). Notably, the swine selected at different time points had similar weights, ages, and other baseline characteristics, ensuring the comparability of results (Figure 5b).

In the *in vivo* study, all animals were successfully treated with IVL or PTA, achieving 100% surgical and technical success rates. No animals died during treatment or follow-up, except those sacrificed per protocol. Blood tests showed that IVL treatment did not increase the incidence of systemic inflammatory responses, affect red blood cell function, or alter platelet counts, indicating that IVL does not induce systemic inflammation or disrupt coagulation-related factors such as platelets. Moreover, IVL treatment did not significantly impact liver or kidney function, circulating lipid levels, glucose levels, or ion concentrations during different follow-up periods (Supplementary Table S2), demonstrating good biosafety.

Follow-up DSA imaging showed that IVL significantly improved the immediate diameter expansion of the treated artery by +27.12 ± 10.23% compared with +13.72 ± 7.66% with the PTA balloon (*n* = 9, *p* = 0.0063; Figure 5c). IVL treatment also significantly reduced the lumen loss rate compared with PTA at 7 days (1.10 ± 0.58% vs. 3.27 ± 0.66%) and 28 days (4.90 ± 1.60% vs. 10.10 ± 1.53%) postoperatively (*p* < 0.05; Figure 5d). These imaging results indicate that IVL treatment achieved greater luminal expansion and significantly reduced lumen loss during follow-up compared to traditional PTA balloons.

Pathological and immunofluorescence staining of arterial specimens confirmed that IVL treatment did not increase inflammation, collagen and polysaccharide-protein synthesis, thrombosis formation, vascular smooth muscle cell loss, necrosis, or thrombosis in the arterial wall at 0, 7, and 28 days postoperatively (Figures 5e–g).

**Figure 6:**
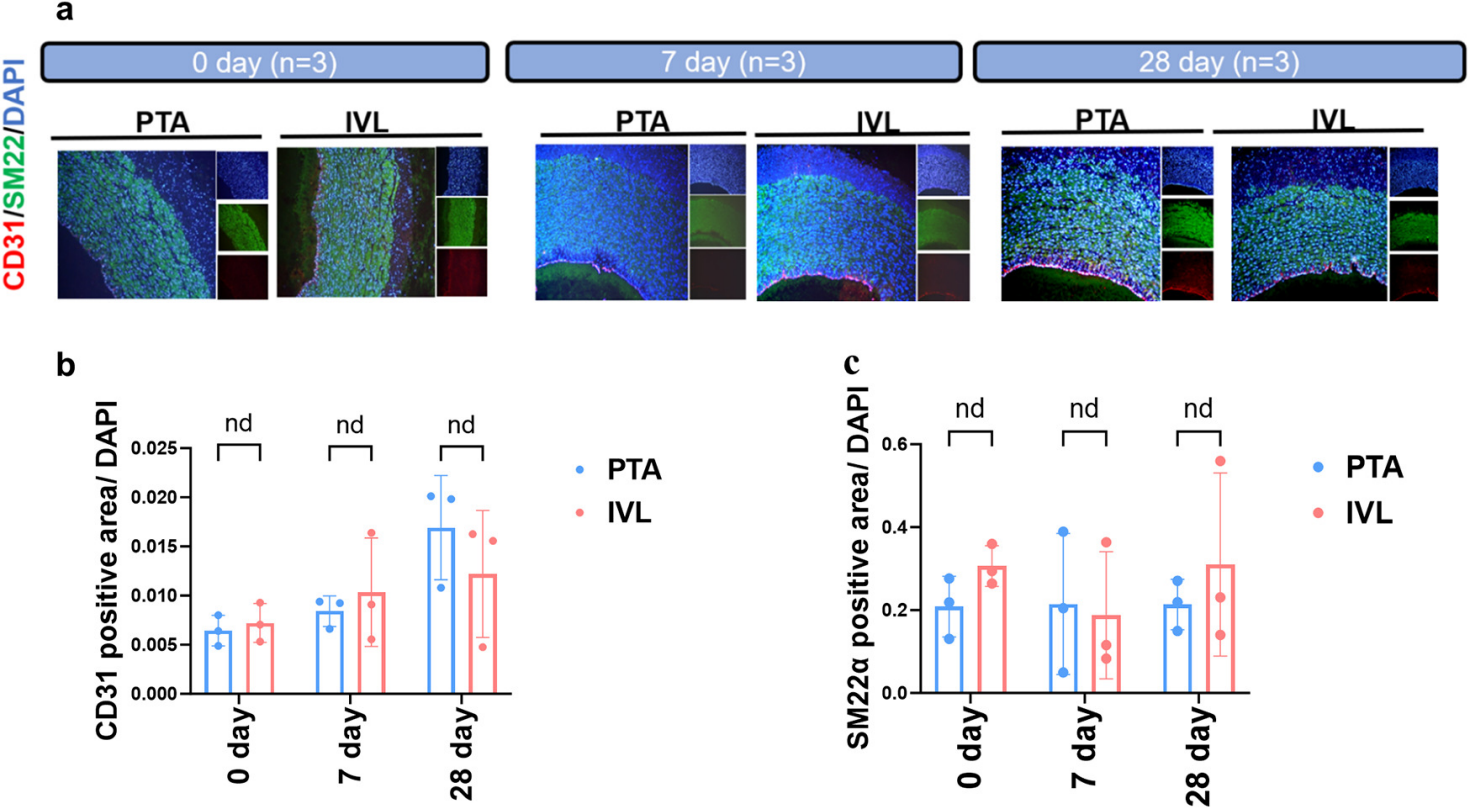
Immunofluorescence staining analysis. **(a)** Immunofluorescence detection of target arteries’ vascular smooth muscle and endothelial cell content; **(b,c)** calculation of vascular smooth muscle and endothelial cell proportion in the target arteries. CD31, marker of endothelial cell; SM22α, marker of vascular smooth muscle cell.

Furthermore, IVL treatment did not reduce the proportion of smooth muscle or endothelial cells (Figures 6a–c). In summary, radiological and pathological findings indicate that the IVL device provides greater luminal diameter expansion without compromising arterial integrity.

## Discussion

This study provides a comprehensive evaluation of a novel IVL system by integrating an *in vitro* gypsum model and *in vivo* animal experiments. In this study, using models with different thicknesses and eccentric calcifications, the IVL device demonstrated favorable therapeutic effects on calcifications with varying characteristics. By testing different oversized ratios, the optimal IVL balloon oversize ratio was identified using an *in vitro* gypsum model. In the *in vivo* study, DSA imaging showed that the IVL device provided better immediate lumen acquisition and a lower follow-up lumen loss rate compared to PTA balloons. These findings suggest that IVL may offer superior immediate postoperative and short-term therapeutic effects for patients with PAD. The *in vitro* study also demonstrated that the IVL device achieved good lumen acquisition without compromising the integrity of endothelial and vascular smooth muscle cells, indicating good biosafety. Overall, the study quantitatively demonstrated the calcification-disrupting efficacy of IVL on different types of calcifications and highlighted its potential to improve clinical outcomes for patients with PAD.

As calcification increases, the risk of vascular complications after treatment (18, 19). Calcification within the tunica media has long posed a challenge in EVT for patients with PAD, as few devices can effectively remove or disrupt calcified plaques embedded deep within the vessel wall (8). Traditional treatments often involve increasing balloon pressure to crack calcification in the tunica media, which may be partially effective but carries a risk of vascular rupture when excessive dilation is applied (20, 21). Although directional atherectomy can more thoroughly address arterial calcification and may be the only EVT method capable of physically removing plaques, a high rate of arterial rupture and other postoperative complications related to the device pose a challenge to its clinical safety (4). Compared with other lesion preparation devices such as atherectomy (4) and ELA (5), IVL offers distinct advantages, particularly for heavily calcified lesions and those located in the tunica media. This makes IVL a valuable addition to the range of lesion preparation tools for PAD, addressing a critical need for treating complex calcified lesions, especially those within the tunica media where direct-contact devices may be less effective or pose higher risks (22).

Intravascular lithotripsy is a novel technique for modifying calcified plaques, utilizing a single-use balloon catheter embedded with lithotripsy emitters. It adopts concepts similar to those of shockwave lithotripsy used for nephrolithiasis, generating pulsatile sonic pressure waves to safely disrupt calcification. The device was first described in the treatment of peripheral arteries in 2016 (23), and gained European and United States Food and Drug Administration approvals in 2018 (24).

Early reports showed that the use of IVL has a consistent reduction in stenoses and low procedural complication rates in the PAD treatment (9, 25). In the recent study, Stefano Fazzini et al. (26). reported the mid-term outcomes of shockwave intravascular lithotripsy in the calcified illiac arteries. The mid-term results of the retrospective cohort study showed that IVL has a high surgical success rate and clinical technical success rate with a low incidence of device adverse events and complications, and has high target artery patency during the 24-month follow-up period. A systematic review and meta-analysis study revealed that the application of IVL can reduce the stenosis of the target artery by 59.31% (95% CI: 53.30%–65.31%), and only 1.25% (95% CI: 0.60%–2.61%) D-grade or above flow restriction dissection can be found after the IVL treatment (24). A prospective clinical study reported that the application of IVL can improve the ankle brachial index (ABI) from 0.74 ± 0.20 to 0.97 ± 0.18 at 30 days (27), and Radaideh et al. (28) reported that the ABI can be increased from 0.7 ± 0.1 to 0.9 ± 0.2 at six months, and the PAD II clinical study revealed the ABI was increased from 0.7 ± 0.2 to 1.0 ± 0.2 at 12 months (11). Clinical cohort study also indicated that the use of IVL can reduce the Rutherford classification from R3 to R0 in PAD patients within a 6 and 12 follow-up period (11). A short-term retrospective study revealed that there were low target lesion revascularisation (TLR) rates within a 12-month follow-up period (11, 28). Furthermore, meta-analysis results also revealed the use of additional stents was only 15.89% (95% CI: 5.22%–39.34%) after IVL treatment (24). These results demonstrated that IVL has good effectiveness and satisfactory safety in reducing the degree of calcified arterial stenosis and improving the treatment efficacy of EVT in severe PAD patients.

Compared with other IVL devices, our IVL device has some characteristics. Firstly, our IVL device has five symmetrically distributed unfocused electrohydraulic emitters. This design generates 360° homogeneous shockwaves (1.0–9.7 MPa) that have deep penetration (up to 2.5 mm) to reach tunica media-embedded calcifications. Furthermore, our IVL has lower operating pressure (4 atm vs. 10 atm in PTA balloons and 6 atm in the shockwave IVL device) minimizes mechanical stress on the vessel wall to avoid barotrauma to the normal tissue (11). These advantages will provide more effective and safe selection of luminal preparation equipment for patients with severe calcified PAD, although the exact clinical efficacy requires further prospective, large-scale, long-term follow-up head-to-head clinical confirmation.

IVL leverages the difference in density between calcified plaques and normal blood vessels, which creates an impedance mismatch in acoustic properties. As a result, tissues with higher density experience greater pressure from non-aggregated low-intensity shockwaves, enabling calcification disruption without direct contact. Although prospective clinical studies (10, 29) have shown that IVL can promote the efficacy of EVT in PAD patients with moderate to severe calcified, the disrupting efficacy of IVL on different characteristic calcifications has not been quantitatively demonstrated. Through this study, we quantitatively described the therapeutic efficacy of IVL on different characteristics of calcified lesions for the first time through an *in vitro* experiment based on a customer-modified gypsum calcification model, revealing the quantitative description of IVL's disruption efficacy on different characteristics of calcified lesions. Our research could provide basic data support for personalized IVL device design for PAD patients.

The previous studies (17) have shown that IVL can alleviate static barotrauma to the treated artery by reducing the working balloon's maximum working pressure. In the present study, our *in vitro* quantitative experiments showed that an over-expansion (target lesion diameter: IVL working balloon diameter) ratio of 1.04:1 was sufficient to provide IVL to disrupt calcified plaques, and excessively increasing the working pressure of the IVL working balloon does not bring additional plaque disruption benefits. This will provide clinical interventional physicians with reference data.

It is worth noting that prospective clinical studies have shown that IVL can improve therapeutic efficacy by promoting the degree of eccentric calcification disruption in coronary arteries (30, 31), indicating that IVL has a good therapeutic effect on eccentric calcification. Similarly, previous studies have shown that eccentric calcification was associated with postoperative re-intervention in peripheral arteries (32). Our *in vitro* quantitative study showed that IVL has a good disruption effect on eccentric calcification, indicating the potential role of IVL in improving the therapeutic efficacy of EVT for peripheral arterial disease with eccentric calcification. However, its exact efficacy still needs to be supported by real-world prospective clinical data. It is worth noting that our gypsum model experiments revealed that IVL efficacy plateaued for calcifications ≥3.0 mm, even with double-frequency treatment, whereas 2.5 mm plaques showed significant improvement. This nonlinear response stems from thickness-dependent energy attenuation. For 2.5 mm calcifications, initial pulses generate microfractures that reduce acoustic impedance; subsequent pulses exploit these defects through stress concentration and cyclic fatigue. In contrast, thicker plaques (≥3.0 mm) dissipate shockwave energy before critical stress thresholds are reached, limiting incremental benefit. This aligns with urological lithotripsy models where successive pulses enhance stone fragmentation (16). These findings highlight the importance of personalized IVL dosing based on calcification morphology—a strategy warranting clinical validation.

In the *in vivo* study, IVL significantly improved immediate lumen acquisition and reduced late lumen loss, leading to better luminal outcomes compared to PTA balloons. Histopathological and immunofluorescence staining analyses showed that IVL did not increase endothelial damage or vascular smooth muscle loss compared to PTA balloons, indicating that IVL has good biosafety, which was similar to the previous IVL animal study (15). Furthermore, the histopathological scoring results showed that the use of IVL did not affect the structure of normal blood vessel walls, and did not lead to more severe negative vascular remodeling after usage. These results indicated that IVL did not affect the structure of normal blood vessel walls, which was consistent with the previous study (15). In our *in vivo* study, the lumen diameter expansion measurements were interpreted strictly as the mechanical compliance response of the healthy artery to the IVL or PTA balloon inflation procedure itself. We measured the immediate increase in lumen diameter relative to the pre-treatment baseline within the treated segment. This assesses the device's ability to achieve acute luminal gain in a compliant vessel, which is a fundamental step before application in calcified, non-compliant vessels. While it does not directly measure “calcification disruption” efficacy (which was the focus of the *in vitro* gypsum model), it provides crucial data on the acute lumen acquisition and potential barotrauma compared to standard PTA. Thus, from our perspective, the current preclinical experiments provide quantitative research data on the immediate acquisition and damage of non-calcified normal swine blood vessels by the IVL device, which will provide a necessary preliminary research basis for the exact effectiveness and safety of IVL in aortic calcification swine models in the future.

While our healthy swine model allowed controlled assessment of IVL's safety profile, we acknowledge it does not fully replicate the complex calcified milieu of human PAD. The absence of medial calcification limits direct extrapolation of efficacy outcomes to diseased human arteries. Future studies using large-animal models with induced medial calcification were warranted to validate therapeutic efficacy in pathophysiological contexts.

This study has several limitations. Firstly, the current preclinical study was based on a small healthy swine animal cohort, which was designed to conform to the 3R principles of experimental animals (33), including Replacement, Reduction, and Refinement, to obtain the most valuable results with the smallest sacrifice of animals. Although experiments in healthy animals have shown that IVL obtains better lumens than PTA, the exact efficacy of IVL in a large animal arterial calcification model with a larger cohort and longer follow-up period needs further exploration. Furthermore, expanding the animal sample size is essential to better assess the actual impact of IVL devices on lumen acquisition. The present study also lacks comparison with other established devices (e.g., shockwave IVL) and other debulking devices, which would be addressed through a head-to-head comparison study in a future study. On the other hand, long-term follow-up experiments based on a large animal arterial calcification model are needed to evaluate the long-term effectiveness and safety of IVL on calcified blood arteries. Also, the current research lacked an accurate evaluation of the effectiveness and safety of this IVL device in real-world PAD patients, as well as a comparison with other debulking devices. It is necessary to perform multicenter, prospective, long-term follow-up clinical trials based on clinical PAD patients to accurately evaluate the effectiveness and safety of current IVL devices for patients with severe calcified lesions, even compared with other debulking devices in a head-to-head comparison study. And this limitation would be addressed in our further study.

Despite these limitations, this multifaceted study provides robust evidence supporting the efficacy and safety of this novel IVL system for treating calcified PAD lesions. The findings from *in vitro* and *in vivo* investigations collectively suggest that this IVL device holds significant potential for improving outcomes in patients with PAD and challenging calcified lesions. Future studies should focus on long-term clinical outcomes, larger patient cohorts, and the development of more physiologically relevant animal models of arterial calcification to further validate these findings and explore the full potential of this IVL technology.

## Conclusion

In conclusion, the IVL device has demonstrated effectiveness and safety as a lumen preparation tool for target artery revascularization.

## Data Availability

The raw data supporting the conclusions of this article will be made available by the authors, without undue reservation.

## References

[1] Criqui MH Aboyans V. Epidemiology of peripheral artery disease. Circ Res. (2015) 116:1509–26. 10.1161/CIRCRESAHA.116.303849 25908725

[2] McDermott MM Ho KJ Alabi O Criqui MH Goodney P Hamburg N Disparities in diagnosis, treatment, and outcomes of peripheral artery disease. J Am Coll Cardiol. (2023) 82:2312–28. 10.1016/j.jacc.2023.09.830 38057074

[3] Gornik HL Aronow HD Goodney PP Arya S Brewster LP Byrd L 2024 ACC/AHA/AACVPR/APMA/ABC/SCAI/SVM/SVN/SVS/SIR/VESS guideline for the management of lower extremity peripheral artery disease: a report of the American college of cardiology/American heart association joint committee on clinical practice guidelines. Circulation. (2024) 149:e1313–410. 10.1161/CIR.0000000000001251 38743805 PMC12782132

[4] Wardle BG Ambler GK Radwan RW Hinchliffe RJ Twine CP. Atherectomy for peripheral arterial disease. Cochrane Database Syst Rev. (2020) 9:CD006680. 10.1002/14651858.CD006680.pub3 32990327 PMC8513671

[5] Geach T. Peripheral artery disease: laser light show–targeting in-stent restenosis in peripheral arteries with excimer laser atherectomy. Nat Rev Cardiol. (2015) 12:63. 10.1038/nrcardio.2014.216 25560374

[6] Sagris M Ktenopoulos N Dimitriadis K Papanikolaou A Beneki E Tzoumas A Efficacy of intravascular lithotripsy (IVL) in coronary stenosis with severe calcification: a systematic review and meta-analysis of 38 studies. Eur Heart J. (2023) 44:710–21. 10.1093/eurheartj/ehad655.2179 38482928

[7] Ali ZA Brinton TJ Hill JM Maehara A Matsumura M Karimi GK Optical coherence tomography characterization of coronary lithoplasty for treatment of calcified lesions: first description. JACC Cardiovasc Imaging. (2017) 10:897–906. 10.1016/j.jcmg.2017.05.012 28797412

[8] Kereiakes DJ Virmani R Hokama JY Illindala U Mena-Hurtado C Holden A Principles of intravascular lithotripsy for calcific plaque modification. JACC Cardiovasc Interv. (2021) 14:1275–92. 10.1016/j.jcin.2021.03.036 34167671

[9] Brodmann M Werner M Brinton TJ Illindala U Lansky A Jaff MR Safety and performance of lithoplasty for treatment of calcified peripheral artery lesions. J Am Coll Cardiol. (2017) 70:908–10. 10.1016/j.jacc.2017.06.022 28797363

[10] Tepe G Brodmann M Werner M Bachinsky W Holden A Zeller T Intravascular lithotripsy for peripheral artery calcification. JACC Cardiovasc Interv. (2021) 14:1352–61. 10.1016/j.jcin.2021.04.010 34167675

[11] Brodmann M Werner M Holden A Tepe G Scheinert D Schwindt A Primary outcomes and mechanism of action of intravascular lithotripsy in calcified, femoropopliteal lesions: results of disrupt PAD II. Catheter Cardiovasc Interv. (2019) 93:335–42. 10.1002/ccd.27943 30474206

[12] Suna G Wojakowski W Lynch M Barallobre-Barreiro J Yin X Mayr U Extracellular matrix proteomics reveals interplay of aggrecan and aggrecanases in vascular remodeling of stented coronary arteries. Circulation. (2018) 137:166–83. 10.1161/CIRCULATIONAHA.116.023381 29030347 PMC5757669

[13] Kaluza GL Raizner AE Mazur W Schulz DG Buergler JM Fajardo LF Long-term effects of intracoronary beta-radiation in balloon- and stent-injured porcine coronary arteries. Circulation. (2001) 103:2108–13. 10.1161/01.cir.103.16.2108 11319203

[14] McKellar SH Thompson JL Garcia-Rinaldi RF MacDonald RJ Sundt TM Schaff HV. Short- and long-term efficacy of aspirin and clopidogrel for thromboprophylaxis for mechanical heart valves: an *in vivo* study in swine. J Thorac Cardiovasc Surg. (2008) 136:908–14. 10.1016/j.jtcvs.2008.01.045 18954629

[15] Liu F Ge Y Rong D Zhu Y Yin J Sun G Injury and healing response of healthy peripheral arterial tissue to intravascular lithotripsy: a prospective animal study. Front Cardiovasc Med. (2022) 9:787973. 10.3389/fcvm.2022.787973 35419438 PMC8995801

[16] Pishchalnikov YA McAteer JA Williams JJ Pishchalnikova IV Vonderhaar RJ. Why stones break better at slow shockwave rates than at fast rates: *in vitro* study with a research electrohydraulic lithotripter. J Endourol. (2006) 20:537–41. 10.1089/end.2006.20.537 16903810 PMC2442574

[17] Ali ZA Nef H Escaned J Werner N Banning AP Hill JM Safety and effectiveness of coronary intravascular lithotripsy for treatment of severely calcified coronary stenoses. Circ Cardiovasc Interv. (2019) 12:e008434. 10.1161/CIRCINTERVENTIONS.119.008434 31553205

[18] Rosenfield K Jaff MR White CJ Rocha-Singh K Mena-Hurtado C Metzger DC Trial of a paclitaxel-coated balloon for femoropopliteal artery disease. N Engl J Med. (2015) 373:145–53. 10.1056/NEJMoa1406235 26106946

[19] Tepe G Laird J Schneider P Brodmann M Krishnan P Micari A Drug-coated balloon versus standard percutaneous transluminal angioplasty for the treatment of superficial femoral and popliteal peripheral artery disease 12-month results from the in. PACT SFA randomized trial. Circulation. (2015) 131:495. 10.1161/CIRCULATIONAHA.114.011004 25472980 PMC4323569

[20] Tsetis D Morgan R Belli A. Cutting balloons for the treatment of vascular stenoses. Eur Radiol. (2006) 16:1675–83. 10.1007/s00330-006-0181-x 16609863

[21] Rabbi JF Kiran RP Gersten G Dudrick SJ Dardik A. Early results with infrainguinal cutting balloon angioplasty limits distal dissection. Ann Vasc Surg. (2004) 18:640–3. 10.1007/s10016-004-0103-9 15599620

[22] Kassimis G Didagelos M De Maria GL Kontogiannis N Karamasis GV Katsikis A Shockwave intravascular lithotripsy for the treatment of severe vascular calcification. Angiology. (2020) 71:677–88. 10.1177/0003319720932455 32567327

[23] Brinton T Brodmann M Werner M Tepe G Holden A Scheinert D Safety and performance of the shockwave medical lithoplasty® system in treating calcified peripheral vascular lesions: 6-month results from the two-phase DISRUPT PAD study. J Am Coll Cardiol. (2016) 68:B314. 10.1016/j.jacc.2016.09.808

[24] Wong CP Chan LP Au DM Chan HWC Chan YC. Efficacy and safety of intravascular lithotripsy in lower extremity peripheral artery disease: a systematic review and meta-analysis. Eur J Vasc Endovasc Surg. (2022) 63:446–56. 10.1016/j.ejvs.2021.10.035 34887206

[25] Brodmann M Schwindt A Argyriou A Gammon R. Safety and feasibility of intravascular lithotripsy for treatment of common femoral artery stenoses. J Endovasc Ther. (2019) 26:283–7. 10.1177/1526602819844998 31006305

[26] Fazzini S Turriziani V Lomazzi C Forcella E Grazioli L Allievi S Mid-term outcomes of shockwave intravascular lithotripsy in the IVLIAC registry for the treatment of calcified iliac occlusive disease. J Vasc Surg. (2025) S0741-5214(25)00960-7. 10.1016/j.jvs.2025.04.025 40268258

[27] Adams G Shammas N Mangalmurti S Bernardo NL Miller WE Soukas PA Intravascular lithotripsy for treatment of calcified lower extremity arterial stenosis: initial analysis of the disrupt PAD III study. J Endovasc Ther. (2020) 27:473–80. 10.1177/1526602820914598 32242768 PMC7288854

[28] Radaideh Q Shammas NW Shammas WJ Shammas GA. Shockwave™ lithoplasty in combination with atherectomy in treating severe calcified femoropopliteal and iliac artery disease: a single-center experience. Cardiovasc Revasc Med. (2021) 22:66–70. 10.1016/j.carrev.2020.06.015 32563711

[29] Lopez-Pena G Musto L Finch SL Brown MJ Davies R Sohrabi S SHOCkwave lithotripsy for patients with peripheral arterial disease: the SHOCC study. J Vasc Soc Great Bri Irel. (2024) 3:140–6. 10.54522/jvsgbi.2024.126

[30] Ali ZA Kereiakes DJ Hill JM Saito S Di Mario C Honton B Impact of calcium eccentricity on the safety and effectiveness of coronary intravascular lithotripsy: pooled analysis from the disrupt CAD studies. Circ Cardiovasc Interv. (2023) 16:e012898. 10.1161/CIRCINTERVENTIONS.123.012898 37847770 PMC10573097

[31] Blachutzik F Honton B Escaned J Hill JM Werner N Banning AP Safety and effectiveness of coronary intravascular lithotripsy in eccentric calcified coronary lesions: a patient-level pooled analysis from the disrupt CAD i and CAD II studies. Clin Res Cardiol. (2021) 110:228–36. 10.1007/s00392-020-01737-3 32948882 PMC7862504

[32] Stavroulakis K Bisdas T Torsello G Tsilimparis N Damerau S Argyriou A. Intravascular lithotripsy and drug-coated balloon angioplasty for severely calcified femoropopliteal arterial disease. J Endovasc Ther. (2023) 30:106–13. 10.1177/15266028221075563 35130782 PMC9896408

[33] Tannenbaum J Bennett BT. Russell and burch’s 3rs then and now: the need for clarity in definition and purpose. J Am Assoc Lab Anim Sci. (2015) 54:120–32. 25836957 PMC4382615

