# Supplementary material for: Quantitative evaluation of IVL: based on the tunica media gypsum calcification model and a prospective animal study
Source: Front Cardiovasc Med. 2025 Jul 11;12:1620232. doi: 10.3389/fcvm.2025.1620232 (PMC12291299; doi:10.3389/fcvm.2025.1620232)
Supplement: Supplementary file 1 [file Table1.doc]

| **Supplementary Table 1 Experimental swine preoperative blood biochemical examination** | | | | | |
| --- | --- | --- | --- | --- | --- |
|  | **Total_animals** | **0_day** | **7_day** | **28_day** | **P-value** |
|  | **(N=9)** | **(N=3)** | **(N=3)** | **(N=3)** |  |
| **Weight(kg)** |  |  |  |  |  |
| Mean (SD) | 41.0 (2.40) | 41.7 (2.08) | 41.0 (1.00) | 40.3 (4.04) | 0.836 |
| Median [Min, Max] | 41.0 [38.0, 45.0] | 41.0 [40.0, 44.0] | 41.0 [40.0, 42.0] | 38.0 [38.0, 45.0] |  |
| **WBC(10^9^/L)** |  |  |  |  |  |
| Mean (SD) | 18.7 (4.84) | 16.4 (3.07) | 19.3 (5.28) | 20.5 (6.55) | 0.623625 |
| Median [Min, Max] | 18.1 [13.1, 27.9] | 16.8 [13.1, 19.2] | 18.1 [14.7, 25.1] | 18.3 [15.4, 27.9] |  |
| **RBC(10^12^/L)** |  |  |  |  |  |
| Mean (SD) | 7.64 (0.673) | 7.69 (1.19) | 7.64 (0.437) | 7.59 (0.446) | 0.987643 |
| Median [Min, Max] | 7.43 [6.68, 9.00] | 7.38 [6.68, 9.00] | 7.68 [7.18, 8.05] | 7.43 [7.24, 8.09] |  |
| **HGB(g/L)** |  |  |  |  |  |
| Mean (SD) | 123 (9.96) | 123 (15.4) | 123 (7.94) | 122 (9.85) | 0.994136 |
| Median [Min, Max] | 126 [105, 133] | 130 [105, 133] | 126 [114, 129] | 119 [114, 133] |  |
| **HCT(%)** |  |  |  |  |  |
| Mean (SD) | 38.6 (3.12) | 38.2 (3.58) | 38.2 (4.15) | 39.5 (2.66) | 0.865805 |
| Median [Min, Max] | 38.7 [33.6, 42.6] | 38.7 [34.4, 41.5] | 39.2 [33.6, 41.7] | 38.1 [37.9, 42.6] |  |
| **MCV(fL)** |  |  |  |  |  |
| Mean (SD) | 50.7 (2.82) | 50.1 (3.39) | 50.0 (3.96) | 52.1 (0.625) | 0.643782 |
| Median [Min, Max] | 51.5 [46.2, 54.4] | 51.5 [46.2, 52.5] | 48.7 [46.8, 54.4] | 52.3 [51.4, 52.6] |  |
| **MCH(pg)** |  |  |  |  |  |
| Mean (SD) | 16.1 (0.831) | 16.1 (1.42) | 16.1 (0.586) | 16.1 (0.635) | 0.995185 |
| Median [Min, Max] | 15.9 [14.8, 17.6] | 15.8 [14.8, 17.6] | 15.9 [15.7, 16.8] | 16.5 [15.4, 16.5] |  |
| **MCHC(g/L)** |  |  |  |  |  |
| Mean (SD) | 318 (13.3) | 321 (15.0) | 324 (15.6) | 309 (8.14) | 0.433746 |
| Median [Min, Max] | 315 [300, 340] | 320 [306, 336] | 322 [309, 340] | 313 [300, 315] |  |
| **PLT(10^9^/L)** |  |  |  |  |  |
| Mean (SD) | 436 (157) | 336 (168) | 436 (72.2) | 537 (185) | 0.328653 |
| Median [Min, Max] | 401 [149, 739] | 385 [149, 474] | 401 [388, 519] | 498 [375, 739] |  |
| **RDW_SD(fL)** |  |  |  |  |  |
| Mean (SD) | 34.1 (2.53) | 33.0 (2.36) | 33.2 (1.71) | 36.1 (2.86) | 0.275869 |
| Median [Min, Max] | 33.8 [30.3, 39.2] | 33.8 [30.3, 34.8] | 33.4 [31.4, 34.8] | 35.4 [33.6, 39.2] |  |
| **RDW_CV(%)** |  |  |  |  |  |
| Mean (SD) | 18.7 (1.41) | 18.2 (2.11) | 18.5 (1.10) | 19.2 (1.21) | 0.729307 |
| Median [Min, Max] | 18.5 [16.0, 20.5] | 18.5 [16.0, 20.2] | 17.9 [17.9, 19.8] | 19.1 [18.1, 20.5] |  |
| **T_BIL(μmol/L)** |  |  |  |  |  |
| Mean (SD) | 0.989 (0.739) | 0.633 (0.289) | 1.00 (0.721) | 1.33 (1.10) | 0.575179 |
| Median [Min, Max] | 0.800 [0.300, 2.60] | 0.800 [0.300, 0.800] | 0.800 [0.400, 1.80] | 0.800 [0.600, 2.60] |  |
| **TP(g/L)** |  |  |  |  |  |
| Mean (SD) | 65.3 (4.12) | 66.3 (7.23) | 65.3 (2.89) | 64.3 (2.08) | 0.8734 |
| Median [Min, Max] | 66.0 [58.0, 71.0] | 70.0 [58.0, 71.0] | 67.0 [62.0, 67.0] | 65.0 [62.0, 66.0] |  |
| **ALB(g/L)** |  |  |  |  |  |
| Mean (SD) | 43.2 (4.36) | 45.5 (6.52) | 42.6 (1.19) | 41.3 (4.26) | 0.542317 |
| Median [Min, Max] | 43.0 [36.9, 49.3] | 49.3 [38.0, 49.3] | 43.0 [41.3, 43.6] | 41.6 [36.9, 45.4] |  |
| **ALT(U/L)** |  |  |  |  |  |
| Mean (SD) | 59.8 (7.84) | 58.7 (6.66) | 61.0 (5.57) | 59.7 (12.9) | 0.950654 |
| Median [Min, Max] | 60.0 [49.0, 74.0] | 62.0 [51.0, 63.0] | 60.0 [56.0, 67.0] | 56.0 [49.0, 74.0] |  |
| **AST(U/L)** |  |  |  |  |  |
| Mean (SD) | 34.0 (9.63) | 37.3 (7.57) | 29.7 (14.2) | 35.0 (8.19) | 0.67018 |
| Median [Min, Max] | 33.0 [21.0, 46.0] | 34.0 [32.0, 46.0] | 22.0 [21.0, 46.0] | 33.0 [28.0, 44.0] |  |
| **GGT(U/L)** |  |  |  |  |  |
| Mean (SD) | 42.4 (16.7) | 51.0 (24.2) | 36.7 (17.8) | 39.7 (6.03) | 0.60515 |
| Median [Min, Max] | 39.0 [21.0, 65.0] | 65.0 [23.0, 65.0] | 33.0 [21.0, 56.0] | 39.0 [34.0, 46.0] |  |
| **ALP(U/L)** |  |  |  |  |  |
| Mean (SD) | 117 (52.6) | 135 (14.2) | 142 (80.3) | 73.0 (9.54) | 0.225967 |
| Median [Min, Max] | 119 [58.0, 218] | 143 [119, 144] | 149 [58.0, 218] | 74.0 [63.0, 82.0] |  |
| **Urea(mmol/L)** |  |  |  |  |  |
| Mean (SD) | 3.11 (0.830) | 3.13 (1.01) | 2.47 (0.153) | 3.73 (0.709) | 0.178194 |
| Median [Min, Max] | 2.60 [2.30, 4.50] | 2.60 [2.50, 4.30] | 2.50 [2.30, 2.60] | 3.60 [3.10, 4.50] |  |
| **Cr(μmol/L)** |  |  |  |  |  |
| Mean (SD) | 145 (20.9) | 161 (18.2) | 138 (13.1) | 135 (25.7) | 0.291534 |
| Median [Min, Max] | 150 [106, 182] | 151 [150, 182] | 134 [128, 153] | 146 [106, 154] |  |
| **GLU(mmol/L)** |  |  |  |  |  |
| Mean (SD) | 6.65 (1.88) | 7.10 (1.48) | 6.32 (1.83) | 6.54 (2.83) | 0.898563 |
| Median [Min, Max] | 7.65 [3.32, 8.65] | 7.92 [5.39, 8.00] | 5.53 [5.01, 8.41] | 7.65 [3.32, 8.65] |  |
| **Tch(mmol/L)** |  |  |  |  |  |
| Mean (SD) | 2.08 (0.237) | 1.97 (0.0351) | 2.03 (0.186) | 2.26 (0.341) | 0.312797 |
| Median [Min, Max] | 2.00 [1.83, 2.64] | 1.97 [1.93, 2.00] | 2.05 [1.83, 2.20] | 2.16 [1.98, 2.64] |  |
| **TG(mmol/L)** |  |  |  |  |  |
| Mean (SD) | 0.279 (0.0804) | 0.247 (0.0208) | 0.240 (0.113) | 0.350 (0.0361) | 0.17418 |
| Median [Min, Max] | 0.300 [0.110, 0.390] | 0.240 [0.230, 0.270] | 0.300 [0.110, 0.310] | 0.340 [0.320, 0.390] |  |
| **CK(U/L)** |  |  |  |  |  |
| Mean (SD) | 702 (344) | 964 (168) | 769 (414) | 372 (23.6) | 0.075952 |
| Median [Min, Max] | 791 [344, 1170] | 879 [855, 1160] | 791 [344, 1170] | 364 [354, 399] |  |
| **K(mmol/L)** |  |  |  |  | 0.18195 |
| Mean (SD) | 3.83 (0.273) | 3.74 (0.282) | 4.07 (0.137) | 3.69 (0.266) |  |
| Median [Min, Max] | 3.95 [3.43, 4.22] | 3.81 [3.43, 3.98] | 4.04 [3.95, 4.22] | 3.62 [3.46, 3.98] |  |
| **Na(mmol/L)** |  |  |  |  |  |
| Mean (SD) | 142 (3.23) | 143 (2.65) | 142 (2.65) | 140 (4.73) | 0.657779 |
| Median [Min, Max] | 142 [135, 145] | 144 [140, 145] | 141 [140, 145] | 142 [135, 144] |  |
| **CL(mmol/L)** |  |  |  |  | 0.599217 |
| Mean (SD) | 99.1 (2.76) | 99.7 (0.577) | 100 (1.00) | 97.7 (4.93) |  |
| Median [Min, Max] | 100 [92.0, 101] | 100 [99.0, 100] | 100 [99.0, 101] | 100 [92.0, 101] |  |
| **Ca(mmol/L)** |  |  |  |  |  |
| Mean (SD) | 2.73 (0.125) | 2.79 (0.0404) | 2.76 (0.0700) | 2.65 (0.200) | 0.417151 |
| Median [Min, Max] | 2.75 [2.45, 2.85] | 2.78 [2.75, 2.83] | 2.73 [2.71, 2.84] | 2.65 [2.45, 2.85] |  |
| **P(mmol/L)** |  |  |  |  |  |
| Mean (SD) | 2.41 (0.195) | 2.42 (0.108) | 2.38 (0.341) | 2.42 (0.150) | 0.963429 |
| Median [Min, Max] | 2.43 [2.14, 2.77] | 2.47 [2.30, 2.50] | 2.23 [2.14, 2.77] | 2.43 [2.27, 2.57] |  |
