# Supplementary material for: Quantitative evaluation of IVL: based on the tunica media gypsum calcification model and a prospective animal study
Source: Front Cardiovasc Med. 2025 Jul 11;12:1620232. doi: 10.3389/fcvm.2025.1620232 (PMC12291299; doi:10.3389/fcvm.2025.1620232)
Supplement: Supplementary file 2 [file Table2.doc]

**Supplementary Table 2 Blood biochemical tests on experimental swine at 7 and 28 days after surgery**

|  |  |  |  |  |  |  |
| --- | --- | --- | --- | --- | --- | --- |
|  | | | | | | |
|  | **7 day** | |  | **28 day** | |  |
|  | **before_operation** | **after_operation** | **P-value** | **before_operation** | **after_operation** | **P-value** |
|  | **(N=3)** | **(N=3)** |  | **(N=3)** | **(N=3)** |  |
| **Weight(kg)** |  |  |  |  |  |  |
| Mean (SD) | 41.0 (1.00) | 42.7 (3.79) | 1.000 | 40.3 (4.04) | 45.7 (3.79) | 0.376 |
| Median [Min, Max] | 41.0 [40.0, 42.0] | 41.0 [40.0, 47.0] |  | 38.0 [38.0, 45.0] | 44.0 [43.0, 50.0] |  |
| **WBC(10^9^/L)** |  |  |  |  |  |  |
| Mean (SD) | 19.3 (5.28) | 12.8 (4.89) | 0.400 | 20.5 (6.55) | 19.8 (8.32) | 1.000 |
| Median [Min, Max] | 18.1 [14.7, 25.1] | 10.4 [9.70, 18.5] |  | 18.3 [15.4, 27.9] | 17.9 [12.6, 28.9] |  |
| **RBC(10^12^/L)** |  |  |  |  |  |  |
| Mean (SD) | 7.64 (0.437) | 6.72 (0.664) | 0.200 | 7.59 (0.446) | 6.88 (0.515) | 0.200 |
| Median [Min, Max] | 7.68 [7.18, 8.05] | 6.40 [6.27, 7.48] |  | 7.43 [7.24, 8.09] | 6.97 [6.33, 7.35] |  |
| **HGB(g/L)** |  |  |  |  |  |  |
| Mean (SD) | 123 (7.94) | 107 (3.00) | 0.100 | 122 (9.85) | 112 (6.35) | 0.261 |
| Median [Min, Max] | 126 [114, 129] | 107 [104, 110] |  | 119 [114, 133] | 108 [108, 119] |  |
| **HCT(%)** |  |  |  |  |  |  |
| Mean (SD) | 38.2 (4.15) | 33.0 (1.26) | 0.200 | 39.5 (2.66) | 34.9 (1.46) | 0.100 |
| Median [Min, Max] | 39.2 [33.6, 41.7] | 32.8 [31.8, 34.3] |  | 38.1 [37.9, 42.6] | 35.1 [33.3, 36.2] |  |
| **MCV(fL)** |  |  |  |  |  |  |
| Mean (SD) | 50.0 (3.96) | 49.3 (2.93) | 1.000 | 52.1 (0.625) | 50.8 (1.68) | 0.507 |
| Median [Min, Max] | 48.7 [46.8, 54.4] | 50.7 [45.9, 51.2] |  | 52.3 [51.4, 52.6] | 50.4 [49.3, 52.6] |  |
| **MCH(pg)** |  |  |  |  |  |  |
| Mean (SD) | 16.1 (0.586) | 16.0 (1.14) | 1.000 | 16.1 (0.635) | 16.2 (0.802) | 1.000 |
| Median [Min, Max] | 15.9 [15.7, 16.8] | 16.5 [14.7, 16.8] |  | 16.5 [15.4, 16.5] | 16.1 [15.4, 17.0] |  |
| **MCHC(g/L)** |  |  |  |  |  |  |
| Mean (SD) | 324 (15.6) | 325 (3.21) | 1.000 | 309 (8.14) | 319 (11.2) | 0.400 |
| Median [Min, Max] | 322 [309, 340] | 326 [321, 327] |  | 313 [300, 315] | 323 [306, 327] |  |
| **PLT(10^9^/L)** |  |  |  |  |  |  |
| Mean (SD) | 436 (72.2) | 387 (42.0) | 1.000 | 537 (185) | 454 (130) | 0.700 |
| Median [Min, Max] | 401 [388, 519] | 408 [339, 415] |  | 498 [375, 739] | 484 [311, 566] |  |
| **RDW_SD(fL)** |  |  |  |  |  |  |
| Mean (SD) | 33.2 (1.71) | 34.3 (0.577) | 0.658 | 36.1 (2.86) | 34.8 (2.14) | 1.000 |
| Median [Min, Max] | 33.4 [31.4, 34.8] | 34.6 [33.6, 34.6] |  | 35.4 [33.6, 39.2] | 35.9 [32.3, 36.1] |  |
| **RDW_CV(%)** |  |  |  |  |  |  |
| Mean (SD) | 18.5 (1.10) | 19.1 (1.37) | 0.376 | 19.2 (1.21) | 19.2 (0.850) | 1.000 |
| Median [Min, Max] | 17.9 [17.9, 19.8] | 18.5 [18.2, 20.7] |  | 19.1 [18.1, 20.5] | 19.2 [18.3, 20.0] |  |
| **T_BIL(μmol/L)** |  |  |  |  |  |  |
| Mean (SD) | 1.00 (0.721) | 2.80 (1.87) | 0.400 | 1.33 (1.10) | 0.567 (0.351) | 0.507 |
| Median [Min, Max] | 0.800 [0.400, 1.80] | 3.40 [0.700, 4.30] |  | 0.800 [0.600, 2.60] | 0.600 [0.200, 0.900] |  |
| **TP(g/L)** |  |  |  |  |  |  |
| Mean (SD) | 65.3 (2.89) | 62.0 (1.73) | 0.369 | 64.3 (2.08) | 61.0 (7.94) | 1.000 |
| Median [Min, Max] | 67.0 [62.0, 67.0] | 63.0 [60.0, 63.0] |  | 65.0 [62.0, 66.0] | 64.0 [52.0, 67.0] |  |
| **ALB(g/L)** |  |  |  |  |  |  |
| Mean (SD) | 42.6 (1.19) | 40.7 (0.462) | 0.077 | 41.3 (4.26) | 38.2 (3.34) | 0.400 |
| Median [Min, Max] | 43.0 [41.3, 43.6] | 40.4 [40.4, 41.2] |  | 41.6 [36.9, 45.4] | 36.6 [35.9, 42.0] |  |
| **ALT(U/L)** |  |  |  |  |  |  |
| Mean (SD) | 61.0 (5.57) | 58.3 (3.06) | 0.700 | 59.7 (12.9) | 63.3 (5.51) | 0.700 |
| Median [Min, Max] | 60.0 [56.0, 67.0] | 59.0 [55.0, 61.0] |  | 56.0 [49.0, 74.0] | 66.0 [57.0, 67.0] |  |
| **AST(U/L)** |  |  |  |  |  |  |
| Mean (SD) | 29.7 (14.2) | 45.3 (11.7) | 0.400 | 35.0 (8.19) | 36.7 (20.5) | 1.000 |
| Median [Min, Max] | 22.0 [21.0, 46.0] | 43.0 [35.0, 58.0] |  | 33.0 [28.0, 44.0] | 42.0 [14.0, 54.0] |  |
| **GGT(U/L)** |  |  |  |  |  |  |
| Mean (SD) | 36.7 (17.8) | 24.7 (8.50) | 0.507 | 39.7 (6.03) | 35.3 (5.86) | 0.400 |
| Median [Min, Max] | 33.0 [21.0, 56.0] | 25.0 [16.0, 33.0] |  | 39.0 [34.0, 46.0] | 33.0 [31.0, 42.0] |  |
| **ALP(U/L)** |  |  |  |  |  |  |
| Mean (SD) | 142 (80.3) | 111 (23.2) | 0.700 | 73.0 (9.54) | 84.3 (16.9) | 0.400 |
| Median [Min, Max] | 149 [58.0, 218] | 120 [85.0, 129] |  | 74.0 [63.0, 82.0] | 92.0 [65.0, 96.0] |  |
| **Urea(mmol/L)** |  |  |  |  |  |  |
| Mean (SD) | 2.47 (0.153) | 3.30 (1.04) | 0.121 | 3.73 (0.709) | 2.87 (0.651) | 0.200 |
| Median [Min, Max] | 2.50 [2.30, 2.60] | 2.80 [2.60, 4.50] |  | 3.60 [3.10, 4.50] | 2.90 [2.20, 3.50] |  |
| **Cr(μmol/L)** |  |  |  |  |  |  |
| Mean (SD) | 138 (13.1) | 151 (27.9) | 1.000 | 135 (25.7) | 109 (18.7) | 0.200 |
| Median [Min, Max] | 134 [128, 153] | 145 [126, 181] |  | 146 [106, 154] | 103 [94.0, 130] |  |
| **GLU(mmol/L)** |  |  |  |  |  |  |
| Mean (SD) | 6.32 (1.83) | 5.77 (0.864) | 1.000 | 6.54 (2.83) | 5.25 (0.713) | 0.700 |
| Median [Min, Max] | 5.53 [5.01, 8.41] | 5.54 [5.05, 6.73] |  | 7.65 [3.32, 8.65] | 5.46 [4.46, 5.84] |  |
| **Tch(mmol/L)** |  |  |  |  |  |  |
| Mean (SD) | 2.03 (0.186) | 2.21 (0.376) | 0.700 | 2.26 (0.341) | 1.66 (0.310) | 0.100 |
| Median [Min, Max] | 2.05 [1.83, 2.20] | 2.13 [1.88, 2.62] |  | 2.16 [1.98, 2.64] | 1.68 [1.34, 1.96] |  |
| **TG(mmol/L)** |  |  |  |  |  |  |
| Mean (SD) | 0.240 (0.113) | 0.330 (0.0200) | 0.121 | 0.350 (0.0361) | 0.440 (0.142) | 0.507 |
| Median [Min, Max] | 0.300 [0.110, 0.310] | 0.330 [0.310, 0.350] |  | 0.340 [0.320, 0.390] | 0.390 [0.330, 0.600] |  |
| **CK(U/L)** |  |  |  |  |  |  |
| Mean (SD) | 769 (414) | 842 (417) | 1.000 | 372 (23.6) | 411 (139) | 0.700 |
| Median [Min, Max] | 791 [344, 1170] | 739 [487, 1300] |  | 364 [354, 399] | 413 [271, 548] |  |
| **K(mmol/L)** |  |  |  |  |  |  |
| Mean (SD) | 4.07 (0.137) | 3.83 (0.378) | 0.400 | 3.69 (0.266) | 3.26 (0.349) | 0.200 |
| Median [Min, Max] | 4.04 [3.95, 4.22] | 3.89 [3.43, 4.18] |  | 3.62 [3.46, 3.98] | 3.42 [2.86, 3.50] |  |
| **NA(mmol/L)** |  |  |  |  |  |  |
| Mean (SD) | 142 (2.65) | 140 (2.08) | 0.825 | 140 (4.73) | 129 (19.4) | 0.507 |
| Median [Min, Max] | 141 [140, 145] | 141 [138, 142] |  | 142 [135, 144] | 139 [107, 142] |  |
| **CL(mmol/L)** |  |  |  |  |  |  |
| Mean (SD) | 100 (1.00) | 97.3 (2.08) | 0.121 | 97.7 (4.93) | 91.7 (14.5) | 0.825 |
| Median [Min, Max] | 100 [99.0, 101] | 98.0 [95.0, 99.0] |  | 100 [92.0, 101] | 99.0 [75.0, 101] |  |
| **CA(mmol/L)** |  |  |  |  |  |  |
| Mean (SD) | 2.76 (0.0700) | 2.77 (0.0577) | 0.500 | 2.65 (0.200) | 2.55 (0.321) | 1.000 |
| Median [Min, Max] | 2.73 [2.71, 2.84] | 2.74 [2.74, 2.84] |  | 2.65 [2.45, 2.85] | 2.68 [2.18, 2.78] |  |
| **P(mmol/L)** |  |  |  |  |  |  |
| Mean (SD) | 2.38 (0.341) | 2.35 (0.146) | 1.000 | 2.42 (0.150) | 2.13 (0.376) | 0.400 |
| Median [Min, Max] | 2.23 [2.14, 2.77] | 2.33 [2.22, 2.51] |  | 2.43 [2.27, 2.57] | 2.32 [1.70, 2.38] |  |
